# Porphyrin accumulation in humans with common dysfunctional variants of *ABCG2*, a porphyrin transporter: potential association with acquired photosensitivity

**DOI:** 10.1007/s13577-021-00534-y

**Published:** 2021-05-19

**Authors:** Masayuki Sakiyama, Hirotaka Matsuo, Yu Toyoda, Yuiko Yonekura, Takahiro Ishikawa, Akiyoshi Nakayama, Toshihide Higashino, Yusuke Kawamura, Norihiro Fujimoto, Nariyoshi Shinomiya, Takahiro Satoh

**Affiliations:** 1 Department of Integrative Physiology and Bio-Nano Medicine, National Defense Medical College, 3-2 Namiki, Tokorozawa, Saitama 359-8513 Japan; 2 Department of Dermatology, National Defense Medical College, 3-2 Namiki, Tokorozawa, Saitama 359-8513 Japan

**Keywords:** ABC transporter, Genetic variations, Photodermatosis, Phototoxicity, Porphyrin transport

## Abstract

Photosensitivity is a skin reaction disorder mediated by phototoxic and/or photoallergic mechanisms. The accumulation of porphyrins is generally considered to induce phototoxicity. ATP-binding cassette subfamily G member 2 (ABCG2) has been identified as a transporter of porphyrins and its common variants—p.Gln126Ter (rs72552713) and p.Gln141Lys (rs2231142)—reportedly decrease the function of porphyrin transport in vitro; however, the physiological importance of ABCG2 as a porphyrin transporter remains to be fully elucidated. We herein investigated whether ABCG2 dysfunction could lead to porphyrin accumulation and photosensitivity in Japanese subjects, and found it to be significantly correlated with erythrocyte protoporphyrin levels (*P* = 0.012). This appears to be the first clinical finding of ABCG2 dysfunction-associated protoporphyrin accumulation in humans. We divided the patients into a chronic actinic dermatosis (CAD) group and a non-CAD group. CAD was diagnosed based on the criteria of reduced minimal erythema doses to ultraviolet B (UVB) and/or ultraviolet A (UVA). The non-CAD group was composed of patients who exhibited normal reactions to UVB and UVA on phototesting, but had histories of recurrent erythema/papules on sun-exposed areas. Estimated ABCG2 function according to *ABCG2* genotypes in the non-CAD group was significantly lower than in the general Japanese population (*P* = 0.045). In contrast, no difference was found in ABCG2 function between the CAD group and the general population, suggesting that ABCG2 dysfunction might be a genetic factor in non-CAD patients with clinical photosensitivity. In this context, genetic dysfunction of ABCG2 might be an overlooked pathological etiology of “photosensitivity of unknown cause.”

## Introduction

Photosensitivity is a disorder of the skin caused by reactions that are mediated by phototoxic and/or photoallergic mechanisms in sun-exposed areas of the body. Phototoxic reactions of this nature, which derive from both environmental and genetic factors, are generally caused by drugs, metabolic disturbances, or genetic disorders [1, 2]. Chronic actinic dermatosis (CAD) is a chronic eczematous skin disease of sun-exposed areas that are sensitive to ultraviolet B (UVB) and/or ultraviolet A (UVA) [3]; however, the molecular mechanisms that cause the disease remain unclear. In some cases, such as polymorphous light eruption (PLE), abnormal erythematous reaction such as from a reduced minimal erythema doses (MED) to UVA and UVB on phototests is often missed, despite the presence of clinical photosensitivity. Given the unknown etiology of such skin disorders, there is a growing need to investigate their underlying molecular mechanisms.

The accumulation of porphyrins is understood to be a trigger for phototoxicity, as seen in the examples of erythropoietic protoporphyria and porphyria cutanea tarda [4, 5]. Membrane transport of porphyrins, an important process in their cellular handling, is reportedly mediated by porphyrin transporters [6]. One of these, a urate transporter ATP-binding cassette subfamily G member 2 (ABCG2, also known as breast cancer resistance protein, which has been well-characterized as a drug transporter [7]), has been identified as transporting some porphyrins [8, 9]. In vitro functional assays have shown that two common variants of ABCG2—p.Gln126Ter (p.Q126X: rs72552713) and p.Gln141Lys (p.Q141K: rs2231142) [10]—decrease the function of porphyrin transport [11]. *Abcg2* knockout mice fed on a diet high in alfalfa exhibit severe phototoxicity on light-exposed skin; this diet-dependent phototoxicity is associated with the accumulation of pheophorbide a (Pa, a dietary chlorophyll breakdown product) [12]. Pa is a type of porphyrin and is an ABCG2 substrate, such as some porphyrins, including protoporphyrin IX, a major porphyrin in the blood. Although previous studies such as the above suggest the involvement of ABCG2 dysfunction in several porphyrin-related phototoxicities in mammals, there is little information available on the effects of ABCG2 dysfunction on porphyrin accumulation and photosensitivity in humans. To gain more insight into this issue, we herein investigated the relationship, in Japanese patients, between *ABCG2* genotypes and photosensitivity that lacks apparent causes.

## Methods

### Study participants

This study was approved by the National Defense Medical College’s institutional ethics committee, and all procedures were performed in accordance with the Declaration of Helsinki. Written informed consent was obtained from each subject participating in this study before DNA samples and erythrocyte protoporphyrin data were collected.

The study participants comprised 23 Japanese patients (13 males and 10 females) with suspected photosensitivity based on their clinical histories and/or symptoms. Mean age (years) and standard deviation were 55.5 ± 20.1. Patients with systemic, metabolic, or genetic disorders, including porphyria, xeroderma pigmentosum, and systemic lupus erythematosus, were excluded. Those with drug-induced photosensitivity and photocontact dermatitis were also excluded. We performed phototests with UVB (280–380 nm) and UVA (300–430 nm) lamps of Dermaray-200 (Toshiba Medical Supply, Tokyo, Japan). Seven patients were diagnosed with CAD based on the criteria of reduced MED to UVB (50 mJ/cm^2^) and/or UVA (10 J/cm^2^). Five CAD patients showed photosensitivity to both UVB and UVA; one CAD patient showed photosensitivity to UVA alone, and another CAD patient showed photosensitivity to UVB alone. The remaining 16 patients exhibited normal reactions to UVB and UVA on phototesting, but had histories of recurrent erythema/papules on sun-exposed areas; therefore, they were grouped as non-CAD patients.

### Genetic analysis

Genomic DNA was extracted from whole peripheral blood cells. Genotyping of two *ABCG2* dysfunctional variants, p.Q126X (c.376C > T) and p.Q141K (c.421C > A), was performed using the TaqMan method (Thermo Fisher Scientific, Waltham, MA, USA) with a LightCycler 480 (Roche Diagnostics, Mannheim, Germany), as previously described [13]. Custom TaqMan assay probes were designed as follows. For p.Q126X, VIC-CCACTAATACTTACTTGTACCAC and FAM-CCACTAATACTTACTTATACCAC; for p.Q141K, VIC-CTGCTGAGAACTGTAAGTT and FAM-CTGCTGAGAACTTTAAGTT. To confirm their genotypes, DNA sequencing analysis was performed with the following primers. For p.Q126X, forward 5′-TGTACAATGAAAAGAGAAAGGTGAG-3′ and reverse 5′-CTGCCTTTTCACATAAGTGTC-3′; for p.Q141K, forward 5′-ATGGAGTTAACTGTCAT TTGC-3′ and reverse 5′-CACGTTCATATTATGTAACAAGCC-3′. Direct sequencing was performed with a 3130xl Genetic Analyzer (Thermo Fisher Scientific).

### Statistical analyses

R software (version 3.1.1: http://www.r-project.org/) was used for all the statistical analysis calculations. Linear regression analysis and the Cochran–Armitage test were performed for the association analyses. The *P* value of the Hardy–Weinberg equilibrium was calculated using the Chi-square test with Yates’ correction. We set the significance threshold to *α* = 0.05.

## Results

The call rates for two variants of *ABCG2* (p.Q126X and p.Q141K) were 100%; these variants were in Hardy–Weinberg equilibrium (*P* > 0.05). Reportedly, p.Q126X is a nonfunctional variant and p.Q141K, which halves the protein levels of ABCG2, is a semi-functional variant for protoporphyrin transport [11]. Since there was no simultaneous presence of the minor alleles of p.Q126X and p.Q141K in one haplotype [10], three haplotypes: *1 (Q126 and Q141, the wild-type with full function), *2 (Q126 and K141), and *3 (X126 and Q141) were defined in this study as previously reported [14]. According to the degree of ABCG2 function based on these haplotypes, all the patients could be divided into the following groups: full function (*1/*1), 3/4 function (mild dysfunction; *1/*2), and ≤ 1/2 function (moderate-to-severe dysfunction; *1/*3, *2/*2, *2/*3, or *3/*3) [14, 15], as shown in Table 1.

**Table 1:** Estimated ABCG2 function based on *ABCG2* genotypes in photosensitive patients and a general Japanese population

Estimated ABCG2 function	Diplotype^a^	CAD patients	Non-CAD photosensitive patients	General Japanese population^b^
Full function	*1/*1	4 (57.1%)	4 (25.0%)	253 (50.6%)
3/4 function	*1/*2	2 (28.6%)	8 (50.0%)	177 (35.4%)
≤ 1/2 function	*2/*2, *3/*1, *3/*2 or *3/*3	1 (14.3%)	4 (25.0%)	70 (14.0%)
Total	–	7 (100%)	16 (100%)	500 (100%)
		*P* = 0.82^c^	*P* = 0.045^c^	Reference [13]

*CAD* chronic actinic dermatosis

^a^*1, *2 and *3 represent haplotypes “C–C” (Q126 and Q141), “C-A” (Q126 and K141) and “T-C” (X126 and Q141) of two dysfunctional variants, p.Q126X (c.376C > T; rs72552713) and p.Q141K (c.421C > A; rs2231142), respectively

^b^Data on the general Japanese population were obtained from our previous report [13]

^c^*P* values were calculated using a trend test (Cochran–Armitage test) compared with the general Japanese population

**Fig. 1:**
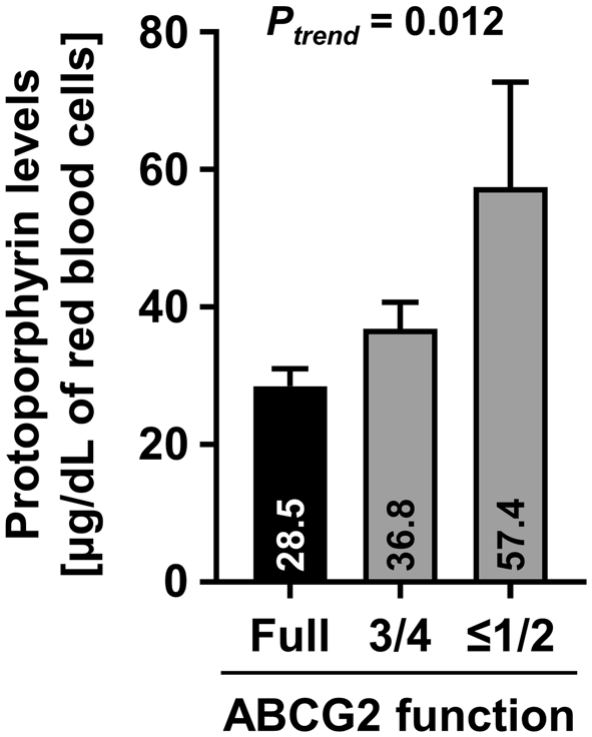
Association between genetic dysfunction of *ABCG2* and higher erythrocyte protoporphyrin levels in photosensitive patients. The degrees of ABCG2 function were determined for 23 patients with photosensitivity based on their genotypes of two common dysfunctional variants of *ABCG2*, p.Q126X and p.Q141K. Data are expressed as mean (which is given within the bars) ± SEM. The *P* value was calculated using linear regression analysis

In the 23 patients with photosensitivity that lacked apparent causes, lower ABCG2 function was significantly correlated with higher erythrocyte protoporphyrin levels (*P* = 0.012; Fig. 1), suggesting that ABCG2 dysfunction can lead to the accumulation of protoporphyrin in humans. We next assessed the association of ABCG2 dysfunction with photosensitivity. For this purpose, we divided the patients into CAD and non-CAD groups, as shown in Table 1. When the non-CAD group was compared with the general Japanese population studied in our previous report [13], the proportions of ABCG2 dysfunctional subjects were significantly different (*P* = 0.045; Table 1). Whereas there were a greater number of ABCG2-dysfunctional subjects in the non-CAD group, this difference was not observed between the CAD patients and the general Japanese population (*P* = 0.82).

## Discussion

In this study, we showed, for the first time to the best of our knowledge, a positive association between genetic dysfunction of ABCG2 and increased erythrocyte protoporphyrin levels in humans, which might result in the potentially higher risk of non-CAD photosensitivity in ABCG2-deficient subjects. Given the intestinal role of ABCG2 as a gatekeeper of ABCG2 substrates [16], together with the fact that ABCG2 also mediates the efflux of protoporphyrin IX from red blood cells into the blood, our findings provide clinical evidence that supports the commonly accepted notion that ABCG2 would regulate porphyrin handling in humans.

This study will also improve our understanding of the genetics-associated risk of diet-induced photosensitivity. Porphyrins are present in natural health supplements, including dried algae. Taking these supplements in large quantities may, therefore, increase the accumulation of porphyrins, which can theoretically raise the risk of photosensitivity in certain individuals, especially those with dysfunctional alleles of *ABCG2*. Ingestion of chlorella tablets has induced phototoxic dermatitis in some individuals with unknown *ABCG2* genotypes: Pa and its ester were identified as photosensitizing agents present in the tablets [17]. We also speculate that an ordinary diet can influence the clinical symptoms of photosensitivity in individuals harboring dysfunctional *ABCG2* alleles. This possibility may, at least partially, explain the negative (non-disease) results of phototests observed in patients in the non-CAD group, despite their histories of the clinical photosensitivity.

Before closing, some limitations warrant mentioning. First, because the non-CAD patient group likely consisted of a heterogeneous population of patients, further studies on more patients with photosensitivity will be needed. Second, we were not able to perform neither phototests with visible light nor photoprovocation tests using repeated exposures or larger single doses of UVB/UVA to address whether the patients could be diagnosed with PLE. Nevertheless, our data suggest that ABCG2 dysfunction may be an overlooked etiology of “photosensitivity of unknown cause” and that it might also affect the severity of phototoxic photosensitivity.

In summary, we report the increased erythrocyte protoporphyrin levels associated with ABCG2 dysfunction. Because genetic variations in *ABCG2* can affect its porphyrin transport activity, our study contribute to a better understanding of the genetic factors that influence individual differences in photosensitivity.
